# Calcium-Mediated Adhesion of Nanomaterials in Reservoir Fluids

**DOI:** 10.1038/s41598-017-11816-7

**Published:** 2017-09-14

**Authors:** Shannon L. Eichmann, Nancy A. Burnham

**Affiliations:** 1 Aramco Services Company: Aramco Research Center - Boston, 400 Technology Square, Cambridge, Massachusetts, 02139 United States of America; 2 Physics and Biomedical Engineering Departments, Worcester Polytechnic Institute, 100 Institute Road, Worcester, Massachusetts, 01609 United States of America; 3 Present Address: Aramco Services Company: Aramco Research Center – Houston, 16300 Park Row Drive, Houston, Texas 77084 United States of America

## Abstract

Globally, a small percentage of oil is recovered from reservoirs using primary and secondary recovery mechanisms, and thus a major focus of the oil industry is toward developing new technologies to increase recovery. Many new technologies utilize surfactants, macromolecules, and even nanoparticles, which are difficult to deploy in harsh reservoir conditions and where failures cause material aggregation and sticking to rock surfaces. To combat these issues, typically material properties are adjusted, but recent studies show that adjusting the dispersing fluid chemistry could have significant impact on material survivability. Herein, the effect of injection fluid salinity and composition on nanomaterial fate is explored using atomic force microscopy (AFM). The results show that the calcium content in reservoir fluids affects the interactions of an AFM tip with a calcite surface, as surrogates for nanomaterials interacting with carbonate reservoir rock. The extreme force sensitivity of AFM provides the ability to elucidate small differences in adhesion at the pico-Newton (pN) level and provides direct information about material survivability. Increasing the calcium content mitigates adhesion at the pN-scale, a possible means to increase nanomaterial survivability in oil reservoirs or to control nanomaterial fate in other aqueous environments.

## Introduction

The ability to produce oil more effectively directly affects the supply of oil worldwide and, as a result, impacts oil prices and thus the cost of goods and services at a global scale^1–5^. Surprisingly, on average only 30% of the oil contained in subsurface reservoirs is recovered during primary and secondary oil production, which include natural pressure drive and water flooding, with recovery of 50–60% being considered exceptional^2, 4^. Thus, there is significant interest in developing new technologies to improve oil recovery through the use of chemicals (i.e. surfactants, polymers, and carbon dioxide) and nano-additives in the later stages of production. At this stage of production, these materials are injected with high salinity water to track flood fronts, determine subsurface flow pathways, or interact with residual oil in the subsurface, which is important to improve reservoir management and increase oil recovery^4, 6–25^. Both classes of materials, however, suffer from a lack of stability in the presence of reservoir rock and high salinity fluids (i.e. seawater and brine) at elevated temperatures, which are conditions common to most oil and gas reservoirs. This lack of stability can lead to serious complications in the subsurface such as pore throat clogging, which reduces fluid permeability and blocks the oil recovery pathway. In addition, the loss of significant amounts of material, even in the absence of pore clogging, can increase costs making these oil recovery efforts uneconomical.

The reservoir conditions present in the Middle East, where Saudi Arabia alone represents ~13% of the oil produced globally^26^ and is pursing oil recoveries up to 70% (double the world average), present a significant challenge to increasing material stability. Specifically, these challenges are tied to the unique combination of extremely high salinities (60 K ppm total dissolved salts (TDS) for seawater (SW) and 120 K ppm TDS for reservoir brine (B)), the presence of carbonate reservoir rock, and temperatures exceeding 100 °C in the subsurface. When studying nanoparticles for reservoir applications, where these materials are used to improve the understanding of subsurface flow pathways or act to mobilize residual oil, material instabilities are evident through nanoparticle-nanoparticle and nanoparticle-surface interactions, which leads to both homo- and hetero-aggregation^9, 13, 17–19, 27, 28^. Materials lacking long-term stability at reservoir conditions could lead to clogged pores within the reservoir or ineffective oil mobilization due to significant quantities of materials being adsorbed by reservoir rock prior to residual oil contact. With a traditional understanding of colloidal stability through Derjaguin, Landau, Verwey, and Overbeek (DLVO) theory, it is expected that particles would strongly adhere at these extreme salinities and that steric stabilizers would be needed to mitigate van der Waals adhesion since the electrostatic repulsion is not present due to high ionic screening^29–31^. In addition, the strength of the interactions should be directly correlated with the overall salinity of the fluids^29–31^. For instance, as the ionic strength increases the Debye screening length for electrostatic repulsion decreases and the dependence is affected by the valence of the ions present in solution. Thus, as the salinity increases (and the Debye length decreases) the overall tip-surface interaction is more affected by van der Waals attraction.

However, it has been recently observed through dynamic light scattering studies, nanoparticle transport, and molecular dynamics simulations that in these harsh reservoir conditions the overall TDS of the reservoir fluid is not a good indicator of sterically stabilized nanoparticle stability^32–36^. Rather, these studies found that the ion composition, along with the amount and type of divalent ions, was related to the fate of the nanomaterials^32–36^. In the study of how ions interact with materials, surfaces, and macromolecules it has been shown that the ions present in solution, such as calcium, can have a direct effect on nanomaterial and macromolecular stability though hydration forces and alteration of the solvation energy of macromolecules, an area of research known as specific ion effects^37–46^. This has been previously observed and utilized in other industries, such as pharmaceuticals and biology, to significantly affect polymer, molecular, and even nanoparticle aggregation behavior, but it is a recent application for the oil industry^27, 38–42, 44–51^.

Atomic Force Microscopy (AFM) is a tool that is most widely known for the ability to provide Angstrom-level resolution of surface topography, and it has been extensively used in physics, materials science, and biology to gain important information about interactions and morphology at the nano-scale^52–57^. In addition to sample topography, AFM is also an extremely sensitive tool to measure tip-surface interactions in both air and fluid environments^54, 57–63^. Through the use of cantilevers with low spring constants and highly sensitive detectors, AFM is capable of measuring interaction forces between materials with pico-Newton (pN) resolution. The ability to measure such weak forces makes AFM an extremely useful tool for understanding molecular and nanomaterial adhesion. Specifically, by directly probing pN-scale interactions AFM can be used to understand subsurface material survivability much faster than would be observable through particle aggregation or static sorption studies that are stochastic and thus can require long studies to observe material instabilities. In the biological community and in recent literature related to reservoir fluids, researchers have studied adhesion at the nanoscale to understand the effects of overall salinity on nanomaterial adhesions, mineral dissolution, molecular adhesion related to protein-protein interactions, and even wettability of reservoir surfaces^35, 51, 56, 59, 64–89^. In such studies, increasing overall salinity increases adhesion, which then plateaus^36, 38, 73, 74, 77, 79–81^. In addition, changes in the interaction forces can be used to link more adhesive areas to mineralogical composition and with changes in wettability^76, 81, 84, 85^. Little work has been done, however, to study i) adhesion at the extremely high salinities present in some reservoirs, and ii) the role of specific ion interactions on nanomaterial adhesion to carbonate (CaCO_3_) rock surfaces in Middle Eastern reservoir fluids^81^.

Herein, AFM is used to measure how the salinity and ion content of reservoir fluids affects the adhesion of materials to calcite surfaces where the AFM tip acts as a surrogate for reservoir nanoparticles (bare or functionalized) or chemical additives (functionalized) and the calcite (CaCO_3_) is a surrogate for limestone reservoir rock. The data presented here were measured using both bare and carboxyl group (-COOH) functionalized AFM tips, which represent both bare nanoparticles and -COOH-coated nanoparticles, respectively. Bare AFM tips present a limited number of hydroxyl (-OH) functional groups, which are of interest when studying the effects of ion content on adhesion properties^32–35, 51, 83, 84, 87, 90, 91^. Thus, AFM tips with a -COOH modification were also used to test the effects of ion content, since this functional group has been shown to interact strongly with calcium ions present in solution and is a common functional group in a wide range of macromolecules used to stabilize nanoparticles^32–35, 51, 83, 84, 87, 90, 91^. The findings presented here have significant implications for nanomaterial transport and stabilization at extreme salinities such as those present in oil reservoirs or other high salinity applications such as nanomaterial fate in the environment, biology, and pharmaceuticals.

## Results and Discussion

**Figure 1:**
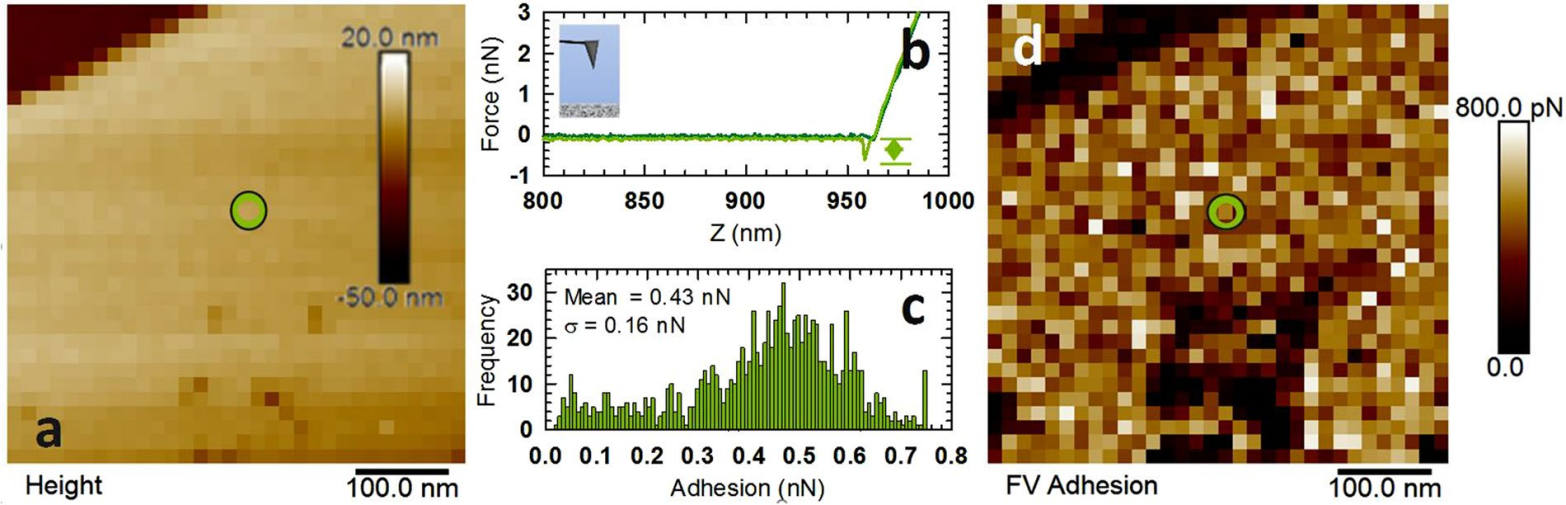
Typical data set from AFM adhesion measurements showing (**a**) calcite topography, (**b**) a typical pair of force curves from the pixel circled in green, (**c**) a histogram of measured adhesion values from 32 × 32 positions, and (**d**) an adhesion map of this area. Adhesion measurements such as these were repeated multiple times in each fluid and then used to study how the chemistry of the fluid affects nanomaterial adhesion.

The adhesion between AFM tips and calcite surfaces in the presence of reservoir fluids was examined to show how changes in the TDS and ion content of these fluids might affect nanomaterial or molecule transport through carbonate oil reservoirs such as those present in the Middle East. Figure 1 shows a typical set of measurement data, where Fig. 1a shows the topography of a 500-nm region from 32 × 32 positions (i.e. 16 nm resolution). Figure 1b shows a pair of force curves from one location on the 32 × 32 grid (location circled in green in Fig. 1a,d), where the darker color is the approach force curve and the lighter color is the force curve for the retracting movement. This treatment of the colors will be used in all subsequent force curve plots. The well depth for the retract curve, indicated by the arrow in Fig. 1b, is measured at each location in the 32 × 32 grid and can then be used to make an adhesion map (Fig. 1d) or plotted as a histogram as shown in Fig. 1c, where the average and standard deviation from each calcite area are calculated.

**Figure 2:**
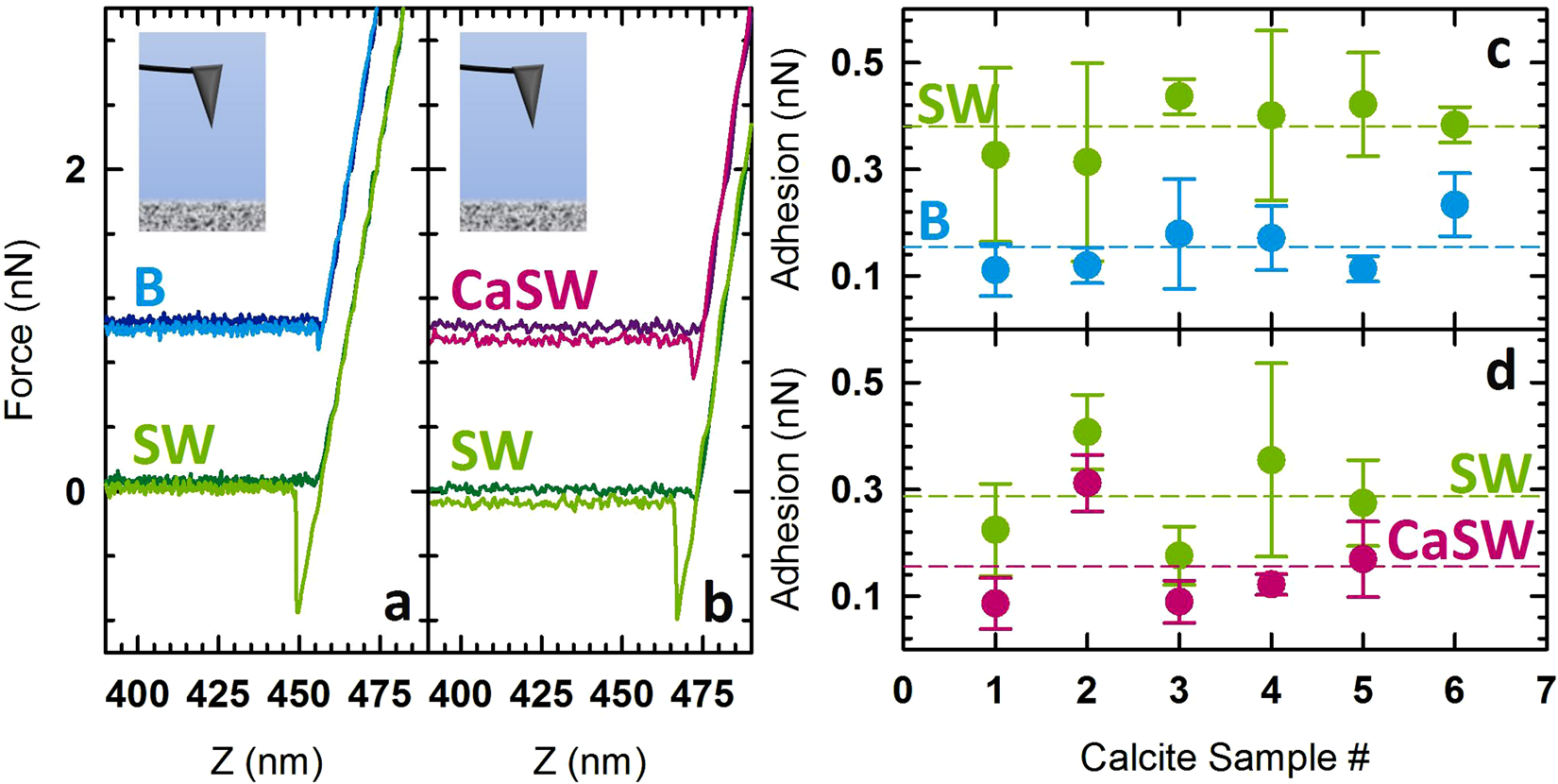
Bare AFM tip data showing (**a**) representative force curves for a bare AFM tip interacting with a calcite surface in two fluids, brine (B) and seawater (SW); (**b**) representative force curves for a bare AFM tip interacting with a calcite surface in two fluids, calcium-doped seawater (CaSW) and SW; (**c**) average adhesion in SW and B for multiple measurements on different calcite samples in B and SW; and (**d**) average adhesion in SW and CaSW for multiple measurements on different calcite samples. In inset in Fig. 2a,b are shown to remind the reader that the AFM tip is bare in these measurements. In SW a bare AFM tip is more strongly adhesive than an AFM tip in B or in CaSW. By doping SW with additional calcium the interaction in B and CaSW is similar.

The first pair of reservoir fluids was selected to demonstrate the differences in adhesion when nanomaterials in a carbonate reservoir experience salinity differences (i.e. overall TDS and ionic composition) due to B and SW. Table 1 shows the composition of these two fluids where the overall molarity of the B is double that of SW. In addition, the SW contains significantly less calcium than the B. Figure 2a shows representative force curves (offset to make all curves visible) from one such experiment with a bare AFM tip. When comparing the retraction curves it can be seen that the adhesion of a bare silicon AFM tip to calcite is considerably higher in SW than in B. This result is surprising given that it is counter to the traditional colloidal understanding based on DLVO theory that at these extremely high salinities all electrostatic repulsion should be screened leaving van der Waals attraction to dominate the interactions. However, this understanding does not take into account the variations in the ion content between B and SW, of which B has a significantly higher concentration of divalent ions and may provide a barrier to nanomaterial adhesion to reservoir surfaces due to hydration forces, as previously reported in other fields^32–34, 45, 46, 50^. In addition, it was previously shown by both experimental and modeling approaches that differences in the Ca^2+^ concentration of these fluids affected particle-particle aggregation for dextran-coated nanoparticles and contributed to dextran adhesion to calcite^32–34^.

**Table 1:** Reservoir fluid composition by concentration of salts added.

Salts	Brine (B) [g/L]	Brine (B) [M]	Seawater (SW) [g/L]	Seawater (SW) [M]	Ca-Doped Seawater (CaSW) [g/L]	Ca-Doped Seawater (CaSW) [M]
NaCl	74.59	1.28	41.04	0.70	41.04	0.70
CaCl_2_·2H_2_O	49.79	0.34	2.39	0.02	7.35	0.05
MgCl_2_·6H_2_O	13.17	0.06	17.65	0.09	17.65	0.09
BaCl_2_	0.01	0.00	0.00	0.00	0.00	0.00
Na_2_SO_4_	0.60	0.00	6.34	0.04	6.34	0.04
NaHCO_3_	0.51	0.01	0.17	0.00	0.17	0.00
Na_2_CO_3_	0.00	0.00	0.00	0.00	0.00	0.00

These previous studies also indicated that increasing the Ca^2+^ concentration in SW to 50 mM (CaCl_2_ saturation reached) would mitigate the particle-particle aggregation and dextran adhesion to calcite^32–34^. With these results in mind, the effect of changing the ionic composition of the SW by doping with additional Ca^2+^ on the adhesion of a bare tip to a calcite surface was tested. Table 1 shows that the overall ionic strength of SW and CaSW where the CaSW is higher due to the additional calcium and contains more divalent ions. Figure 2b shows representative AFM curves (offset to make all curves visible) for the measurements, which shows that by adjusting the Ca^2+^ concentration the adhesion can be mitigated. This finding may have significant implications related to adjusting salinities for EOR applications where chemicals (i.e. surfactants, polymers, and carbon dioxide) and nano-additives could be leveraged to increase oil recovery^15, 16, 32–34^. Figure 2c,d show the average and standard deviation over the maps where each point is the average of 5-10 mapped areas and the error bars are one standard deviation of the data. Additionally, each point indicates a measurement for a different calcite sample and new AFM tips were used each time. Finally, the horizontal dashed lines in 2c and 2d are the average of all these data for each fluid. These data show that a bare AFM tip is more adhesive toward a calcite surface in SW than in B or CaSW and that doping SW with additional Ca^2+^ mitigates the adhesion, bringing the adhesion in SW down to similar levels as in B.

**Figure 3:**
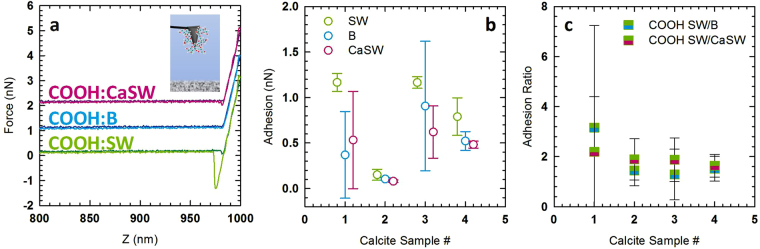
Carboxylated (-COOH) AFM tip data showing (**a**) representative force curves for the interaction with calcite in the presence of SW, B, and CaSW, (**b**) for each fluid (SW, B, or CaSW) on each calcite sample (twelve total data points shown), the average and standard deviation are from three locations on the calcite (the data are offset so that each fluid is visible), and (**c**) the average and standard deviation of the ratio of the adhesion in SW versus B and SW versus CaSW. The inset in Fig. 3a is added to remind the reader that -COOH functionalized tip were used. For -COOH functionalized AFM tips the interactions are stronger than the interactions with the bare tips, and the trends in the interactions are consistent with the bare AFM tip cases where the strongest adhesion is present in SW, while in B and CaSW is adhesion is weaker but similar.

In previous literature on specific ion effects, it has been shown that -COOH and -OH groups bind ions with differing specificity and thus develop a hydration layer that mitigates molecular adhesion, nanoparticle adhesion, and aggregation^32–35, 51, 83, 84, 87, 90, 91^. Thus, to study the hypothesis that the adhesion differences seen in these fluids are due to hydration layers, -COOH functionalized AFM tips were used. Since -COOH has been shown to interact strongly with Ca^2+^ ions through specific ion effects it would provide a stronger interaction with the calcite surface and better distinguish the effects of changing the ion composition in these fluids. Figure 3a shows sample force curves for a -COOH functionalized tip interacting with a calcite surface in the three reservoir fluids, B, SW and CaSW, studied here. Figure 3b shows the adhesion for each fluid (SW, B, or CaSW) for each calcite sample, where each point is the average of three 32 × 32 measurements on different areas of the four calcite samples. Once again, the adhesion is significantly higher in SW than in B or CaSW. In addition to observing this trend, the overall strength of the adhesion is significantly higher for the -COOH modified AFM tips, which would be expected if these functional groups were indeed binding Ca^2+^. At first glance, the data for calcite sample 2 seem low. However, Fig. 3c shows the ratio of the average adhesion in these fluids (SW/B and SW/CaSW) for each of the calcite samples. When normalized, the data for calcite sample 2 are consistent with the other samples; the difference in the adhesion for this sample could be due to poor chemical modification or tip radius effects. Given that the magnitude of the adhesion is on par with that of the bare AFM tips, it is likely that the lower adhesion for calcite sample 2 is related to poor chemical functionalization but tip radius effects cannot be fully discounted.

**Figure 4:**
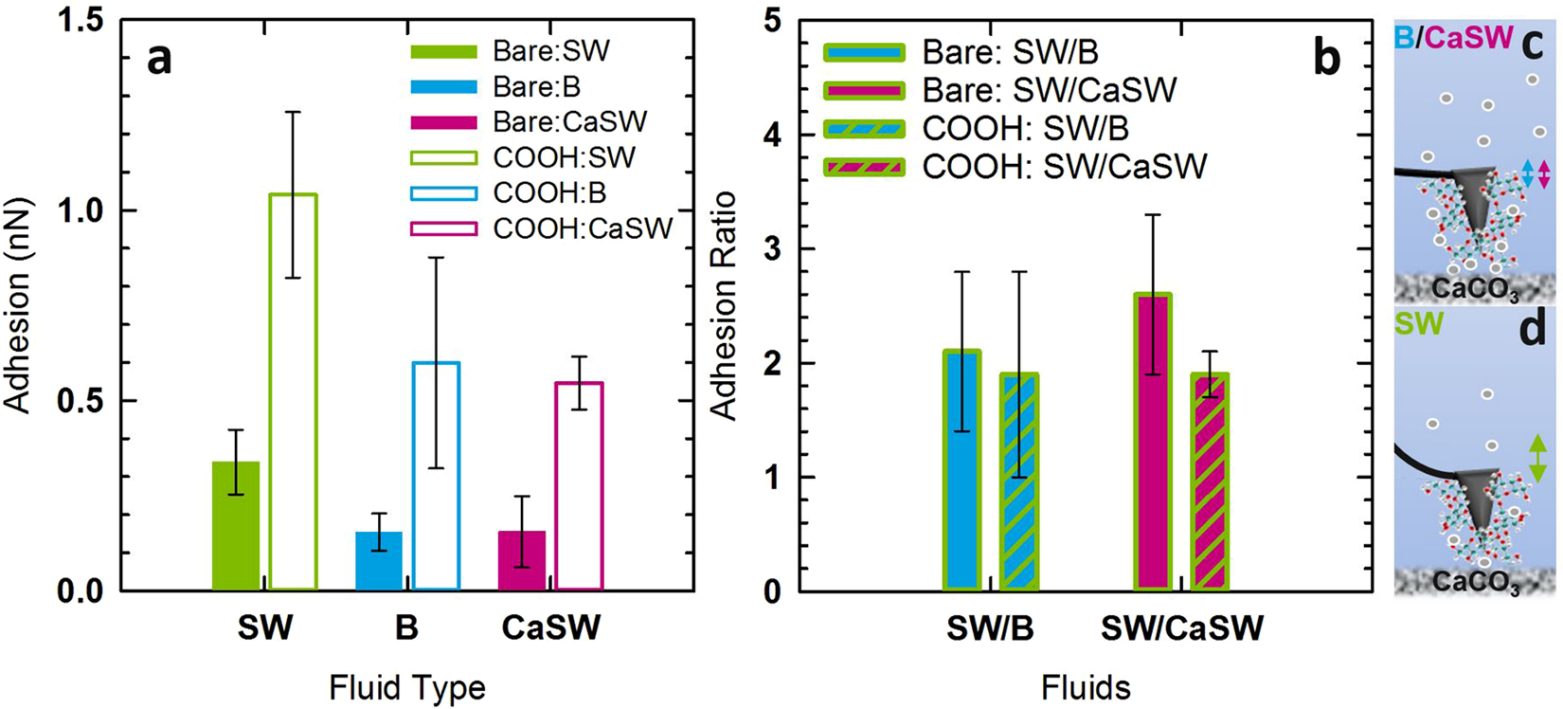
Compiled data for the bare and carboxylated AFM tips interacting with the calcite samples in SW, B, and CaSW. (**a**) The average of all measurements in each fluid for bare and -COOH-coated AFM tips. The error bar is one standard deviation. In both cases the adhesion is lower in B than in SW. By adding Ca^2+^ to the SW to produce CaSW the adhesion is mediated. (**b**) The average of the ratio of the measured adhesion for each tip-calcite pair in each fluid showing that differences in tip radius were not a factor in these matched pairs or triplet experiments. (**c**,**d**) Schematics of how increasing Ca^2+^ in solution might mitigate the adhesion of the tip to the exposed calcium of the calcite surface by closely associating with the -COOH groups present on the functionalized tips. A similar association would be expected for the -OH groups on the bare tips.

For direct comparison, Fig. 4a shows the average and standard deviation of the adhesion from all measurements on the calcite samples. The filled bars are the bare AFM tip data and the white bars are the -COOH functionalized tip data. Here, for both the bare and -COOH functionalized AFM tips, the interactions in SW are more adhesive than in B and CaSW. In addition, the results show that doping SW with additional Ca^2+^ mediates the tip-calcite interactions making the adhesion on par with that of B. To demonstrate that the lack of independently measured tip radius did not significantly impact the data overall, the average and standard deviation of the ratio of the adhesion in the matched pairs and matched triplet experiments are shown in Fig. 4b. After normalizing the data such that the tip radius is no longer a parameter; all data fall within one standard deviation. Given that this trend is the same for both coated and uncoated AFM tips, it is believed that with a calcite surface in SW the tip is attracted to the Ca^2+^ in the calcite sample because the amount of Ca^2+^ in the solution is not high enough to mitigate this interaction in SW (see Fig. 4d). In cases where there is excess Ca^2+^ in solution, such as is the case with B and CaSW fluids, the Ca^2+^ in solution binds to these adhesive sites and mitigates the interactions (see Fig. 4c).

Based on traditional colloidal theory, nanomaterials would be expected to adhere to calcite surfaces with varying strength where the higher salinity in B (120 K ppm) would produce stronger adhesion than the lower salinity in SW (60 K ppm). Through simulation and particle aggregation studies using harsh conditions similar to those in the reservoir, however, it was found that the Ca^2+^ concentrations in these fluids modified nanomaterial-nanomaterial interactions through hydration layers that mediated the interactions, thus making the adhesion in SW higher than in B^32–34^. Here, the results showed that this is similarly true for nanomaterial-calcite interactions. For both bare and -COOH-modified AFM tips, the adhesion in SW was stronger than the adhesion in B or CaSW, and by adding the proper amount of Ca^2+^ to the SW, the interactions in CaSW could be matched to that of B.

While the effects of temperature were not explored in this work, there are a number of possible effects that could be expected as temperature increases for nanomaterials and macromolecules in fluids, as is the case in actual reservoirs. Firstly, for a dispersed system the increase in temperature would increase the likelihood of particle-particle or molecule surface collisions thus increasing the rate of aggregation or adsorption. For cases where macromolecules are present as either an EOR additive or as a stabilizing nanoparticle coating, increasing temperatures may lead to conformational changes or macromolecular decomposition. Changes in the molecular structure could lead to increased adsorption due to changes in the exposed functional groups, loss of steric functionality, or tighter binding of the specific ions present. Finally, for the case of AFM measurements, if the affinity of the AFM tip (either bare or -COOH coated) toward the specific ions present in these reservoir fluids were to increase this could result in an overall decrease in the magnitude of the tip-calcite adhesion forces, but all else being equal the relative trends observed here would likely be maintained.

These findings have implications related to oil recovery applications in cases where producing low-salinity water is not ideal or financially restrictive, such as in the Middle East where sources of fresh water are not available. When SW is the main injection fluid for waterflooding or chemical EOR, it may be more feasible to design the materials to be stable in high salinity conditions and then add a small amount of additional ions to mitigate adhesion toward carbonate (CaCO_3_) reservoir rock. This could improve material survivability by reducing losses in the reservoir and allowing for longer transport, thus improving overall material function, reducing the amount of material needed, and reducing overall cost.

## Materials and Methods

### Fluids

Table 1 shows the salt types and masses (Fisher Scientific, Waltham, MA) added to produce the base reservoir fluids of brine (B) and seawater (SW). These fluids are made by slowly adding each salt to deionized water while stirring. At each step the salt is allowed to dissolve prior to subsequent additions. After mixing all salts and stirring for several hours, the solution is filtered and used. For calcium-doped seawater (CaSW) the same recipe was used to produce the base SW solution but the calcium content was adjusted to 50 mM Ca^2+^.

### AFM Probes

Bare AFM probes with a silicon nitride cantilever and a sharp silicon tip were purchased from Bruker AFM Probes (SNL-10). The cantilever with a nominal spring constant of 0.12 N/m was used for these measurements and all other cantilevers were removed from the chip prior to use. Carboxyl-group functionalized (-COOH) AFM tips with a nominal cantilever spring constant of 0.12 N/m were purchased from NovaScan (Ames, IA).

Prior to each experiment the cantilever was calibrated in air by first updating the deflection sensitivity using a stiff surface and then measuring the spring constant by a thermal tune. The tip radius was not measured in these experiments due to the inability to use a tip-check substrate in fluids. With this in mind, daily experiments were performed where the same tip was used in two or three fluids on the same calcite sample. Repeated measurements were performed with a new tip-calcite pair each time. The data are presented in pairs or triplets corresponding to each experimental run in both un-normalized and normalized form. In cases where obvious evidence of tip changes was observed (i.e. steadily increasing adhesion, significantly changing topographic resolution, etc.) the measurement was deemed unsuitable and either stopped prematurely or discarded upon careful review.

### AFM Measurements

Large calcite (CaCO_3_) samples were purchased from Fisher Scientific and then broken into smaller pieces. Small pieces [typically < (5 L × 5 W × 5 H) mm] of calcite that were smooth (by eye) and possessed a relatively flat surface were chosen as the calcite sample for each AFM measurement. A day prior to starting an experiment a calcite sample was attached to the sample holder using epoxy (Loctite Quickset Epoxy or TW Polymer Waterproof Epoxy) and allowed to set overnight to ensure full curing. For each daily experiment, a new AFM cantilever was loaded in the instrument and calibrated as described above in air, then the calibration sample was replaced with the sample holder containing the calcite sample. Next, enough of the desired fluid (B, SW, or CaSW; filtered with 0.8 µm filter just prior to use) was added to the calcite sample to fully cover the calcite (typically 3–5 mL) and exchanged three times to rinse any large dust from the sample holder or loose calcite debris that might interfere with the measurement. Upon the third addition the fluid was left in the sample chamber and the AFM probe (either bare or -COOH functionalized) was submerged in the fluid and left for 30 minutes to equilibrate. After the equilibration time, the deflection sensitivity was updated prior to beginning the first measurement in each fluid. The deflection sensitivity varied by as much as 20% throughout the day, related to both drift at the detector and repositioning of the laser on the cantilever when changing positions or fluids. To maintain accurate force values, the deflection sensitivity was checked periodically and updated as needed. After data collection in one fluid was complete, it was removed with a pipette and then the new fluid was dispensed and rinsed three times. Then the AFM probe was submerged and allowed to equilibrate again for 30 minutes prior to beginning the next set of measurements.

These experiments were performed on a Bruker Dimension Icon AFM using traditional force-volume mode. A comprehensive overview of AFM and the modes of operation can be found in previous literature^52, 53, 55, 57, 92^. Briefly, the deflection (*x(Z)*) is measured as an AFM cantilever with a known spring constant (*k)* moves perpendicularly to a surface at known distances (Z) from the sample. At each point along this perpendicular movement the interaction force (*F(Z)*) between the sample and the AFM tip is calculated based on Hooke’s Law as $F(Z)=-kx(Z)$ producing a pair of force curves, one upon approach and another upon retraction, for each position above the sample. Next, the tip is rastered above the sample to collect similar force curves at multiple locations on the sample at the desired sampling frequency. Finally, the adhesion at each location on the sample is measured as the depth of the well in the retraction curve. All data were analyzed with the NanoScope Analysis software, which was purchased from Bruker.

For measurements with bare AFM tips, only two fluids were tested per calcite sample and three to five positions on the calcite sample were measured prior to switching the fluid. Additionally, in the initial measurements comparing the adhesion of the bare AFM tip to the calcite surface in B and SW the calcite sample was stored overnight in deionized water. Then, on the next day, the measurements were repeated with a new AFM tip with each fluid on the same piece of calcite rendering a total of five to ten measurements in each fluid, which were averaged to obtain the average adhesion for that calcite sample in each fluid. After confirming the repeatability in this way, the subsequent measurements for bare AFM tips in SW versus CaSW were collected at five different positions on the calcite surface in each fluid and averaged to determine the average adhesion for those fluids. This process was repeated for five to ten individual calcite samples for the B versus SW comparison and an additional five to ten individual calcite samples for the SW versus CaSW comparisons for bare AFM tips. Finally, functionalized (-COOH) tips were used to confirm the effects seen with bare AFM tips. Given the general reproducibility of the data obtained for bare AFM tips in all three fluids, fewer measurements were taken with the (-COOH) tips in each fluid to allow all three fluids to be tested in one day while using one AFM tip and one calcite sample. By using the same tip-calcite pair for all fluids, demonstrating reproducibility over several tip-calcite pairs, and normalizing the results, differences in tip radii are not a concern for these measurements.

### Data availability

The data sets generated during and/or analyzed during the current study are not publicly available due to the corresponding author’s corporate affiliation but are available from the corresponding author on reasonable request.
